# Vitamin D protects dopaminergic neurons against neuroinflammation and oxidative stress in hemiparkinsonian rats

**DOI:** 10.1186/s12974-018-1266-6

**Published:** 2018-08-31

**Authors:** Ludmila A R Lima, Maria Janice P Lopes, Roberta O Costa, Francisco Arnaldo V Lima, Kelly Rose T Neves, Iana B F Calou, Geanne M Andrade, Glauce S B Viana

**Affiliations:** 1 Faculty of Medicine, Federal University of Ceará (UFC), Rua Barbosa de Freitas, 130/1100, Fortaleza, CE 60170-020 Brazil; 2 Faculty of Medicine Estácio of Juazeiro do Norte (Estácio/FMJ), Juazeiro do Norte, Brazil; 3 Federal University of Piauí (UFPI), Picos, Brazil

**Keywords:** Parkinson’s disease and neurodegeneration, Vitamin D, Oxidative stress, Neuroinflammation, Vitamin D receptors

## Abstract

**Background:**

The deficiency in 1α, 25-dihydroxyvitamin D3 (VD3) seems to increase the risk for neurodegenerative pathologies, including Parkinson’s disease (PD). The majority of its actions are mediated by the transcription factor, VD3 receptor (VD3R).

**Methods:**

The neuroprotective effects of VD3 were investigated on a PD model. Male Wistar rats were divided into the following groups: sham-operated (SO), 6-OHDA-lesioned (non-treated), and 6-OHDA-lesioned and treated with VD3 (7 days before the lesion, pre-treatment or for 14 days after the 6-OHDA striatal lesion, post-treatment). Afterwards, the animals were subjected to behavioral tests and euthanized for striatal neurochemical and immunohistochemical assays. The data were analyzed by ANOVA and the Tukey test and considered significant for *p* < 0.05.

**Results:**

We showed that pre- or post-treatments with VD3 reversed behavioral changes and improved the decreased DA contents of the 6-OHDA group. In addition, VD3 reduced the oxidative stress, increased (TH and DAT), and reduced (TNF-alpha) immunostainings in the lesioned striata. While significant decreases in VD3R immunoreactivity were observed after the 6-OHDA lesion, these changes were blocked after VD3 pre- or post-treatments. We showed that VD3 offers neuroprotection, decreasing behavioral changes, DA depletion, and oxidative stress. In addition, it reverses partially or completely TH, DAT, TNF-alpha, and VD3R decreases of immunoreactivities in the non-treated 6-OHDA group.

**Conclusions:**

Taken together, VD3 effects could result from its anti-inflammatory and antioxidant actions and from its actions on VD3R. These findings should stimulate translational research towards the VD3 potential for prevention or treatment of neurodegenerative diseases, as PD.

**Electronic supplementary material:**

The online version of this article (10.1186/s12974-018-1266-6) contains supplementary material, which is available to authorized users.

## Background

Parkinson’s disease (PD) is a consequence of dopaminergic neurodegeneration in the s*ubstantia nigra pars compacta* (*SNpc*). The cardinal signs of the disease are bradykinesia, muscle rigidity, rest tremor, and postural instability. The causes of the beginning of neuronal death are still unclear. Nevertheless, the already known misfolded proteins, specifically α-synuclein, and the mitochondrial dysfunction observed in PD patients create a favorable environment to oxidative stress and neuroinflammation, nurturing a cascade of events that amplify the death signal [1]. The major approaches to PD therapy remain to be the restoration of dopaminergic transmission in basal ganglia, since clinically effective neuroprotective agents that may be able to slow the degeneration of nigral dopaminergic neurons, have not been identified yet [2].

There is therefore a critical need for new drugs and drug targets focusing on the protection of dopaminergic neurons from degeneration [3]. Thus, the choice of a single target against the complex and poorly understood pathophysiology of PD has been shown inefficient [4], what guides the search for new drugs with pleotropic properties, as vitamin D (VD3). Appropriate levels of VD3, whose active metabolite is 1α,25-dihydroxy-vitamin D or simply calcitriol, are essential for the normal neuronal function, such as immunomodulation and detoxification processes [5]. VD3 shows a potent antioxidant activity, being able to reduce lipid peroxidation and to improve brain enzyme activity. A decline in VD3 levels will lead to a dysregulation in ROS and Ca^2+^ signaling pathways and, in some neurodegenerative pathologies as Alzheimer’s disease (AD), it might initiate beta-amyloid formation which progresses to neuronal death and dementia [6, 7]. Some clinical studies point out to the insufficient levels of vitamin D in patients with PD, as well as the relationship between this degree of deficiency and the severity of the disease [8–10].

The mechanism of VD3 action involves the binding of the activated VD3 receptor/retinoic X receptor (VD3R/RXR) heterodimeric complex to specific DNA sequences, that controls gene expression [11, 12]. This VD3 action probably alters genes involved in glutamatergic and GABAergic neurotransmissions, calcium regulation, as well as neurotrophic factors and genes involved in neuroprotection [13]. VD3 deficiency may have a negative influence on critical processes necessary for brain function, as neurotransmission, synapse formation, synaptic plasticity, and dendritic arborization [14]. Furthermore, VD3 deficiency does act as a risk factor for PD development [15].

6-Hydroxydopamine (6-OHDA) when injected into the striatum has been shown to cause a retrograde progressive loss of dopaminergic cell bodies in the SNpc, providing a time window that allows for the testing of new therapeutic options [16]. The ability to protect the dopaminergic neurons, mediated by VD3, has already been demonstrated, however the mechanism involved has not been fully elucidated. In addition, studies that provide robustness to the theory of neuroprotective activity of vitamin D still need to be carried out.

In this context, and knowing that VD3 crosses the blood brain barrier [17], the present study was designed to examine, by means of behavioral, neurochemical and immunohistochemical assays, the potential capacity of VD3 to protect nigrostriatal DA neurons against a partial lesion induced by the unilateral striatal injection of 6-OHDA. In addition, the participation of the brain VD3 receptor (VD3R) in this experimental model of PD was also investigated.

## Methods

### Drugs and reagents

Vitamin D (DePura, colecalciferol, VD3) was purchased from Sanofi, São Paulo, SP, Brazil. Vitamin D (VD3) was suspended in an aqueous solution of 1% DMSO before use. 6-Hydroxydopamine, apomorphine and standards for HPLC measurements were purchased from Sigma-Aldrich, Saint Louis, MO, USA. Ketamine (Vetanercol) and xylazine (Kensol) were from König Laboratory, Santana de Parnaíba, SP, Brazil. Antibodies were from Merck, NJ, USA, Santa Cruz, CA, USA, or from Abcam, CA, USA. All other reagents were of analytical grade.

### Animals

Male Wistar rats (250 g) were housed in a 12-h light/12-h dark cycle, with water and standard food ad libitum*.* The project was approved by the Institutional Committee for Animal Experimentation of the Federal University of Ceará, under the no. 107/2015.

### Experimental design

The animals were divided into four experimental groups (ranging from 4 to 10 animals each): Sham operated (SO), non-treated 6-OHDA, and 6-OHDA treated with 1 μg/kg VD3, for 7 days before surgery (pre-treatment) or for 14 days (post-treatment) after the surgery (6-OHDA+VD3). The SO and the non-treated 6-OHDA groups were treated daily, by gavage with 0.2 mL distilled water. The SO group was submitted to surgery with the infusion of saline in the right striatum. Fourteen days after surgery, the animals from all groups were submitted to behavioral tests and, shortly thereafter, euthanized for neurochemical and immunohistochemistry studies.

### Stereotaxic surgery and 6-OHDA lesion

For screening new therapeutic alternatives, animal models of Parkinson’s disease are widely used. The mostly used animal model of PD consists in the 6-OHDA injection into the rodent striatum. This neurotoxin acts in a retrograde way and is taken up into nigral dopaminergic neurons, initiating cell death by oxidative stress, within a few days [18]. For the experiment, the animals were anesthetized with ketamine (80 mg/kg, i.p.) and xylazine (20 mg/kg, i.p.). Before being placed on the stereotaxic apparatus, the animals were submitted to head trichotomy. A Hamilton syringe was used to inject 6-OHDA in two points (1 μL each, corresponding to a total of 12 μg 6-OHDA, dissolved in saline, containing 0.2% ascorbic acid) into the right striatum. For that, a hole was performed with a hand drill. The bregma was the reference point for the following coordinates, which ensure striatal access: first point: AP, + 0.5; LL, − 2.5; DV, − 5.0; second point: AP, − 0.5; LL, − 3.7; DV, − 6.5, according to the Paxinos and Watson Atlas (2005). For diffusion of 6-OHDA, the needle was left in place for 5 min before being slowly withdrawn.

### Behavioral tests

#### Open field test

This test was performed in order to evaluate the exploratory behavior, as an index of spontaneous motor activity. The apparatus (50 cm × 50 cm × 30 cm, length, width, and height, respectively) was divided into four quadrants of equal size and illuminated by a red light. Each rat was placed in the center of the open field, and the number of crossings was registered for 5 min. After each test, the arena was cleaned with a solution of 70% alcohol [19].

#### Rotational behavior induced by apomorphine

The unilateral injection of 6-OHDA into the striatum triggers motor asymmetry, easily observed after administration of a dopaminergic agonist, such as apomorphine. The motor behavior is characterized by contralateral rotations, in this sensitive test for measuring the degree of striatal lesions [20]. Contralateral rotations for 60 min were counted after the rats were subcutaneously (s.c.) injected with apomorphine, 1 mg/kg.

#### Forced swimming test

This test is based on the observation that when the animals are subjected to a stressful situation, with no possibility for escaping, they adopt a posture of immobility after an initial period of agitation. The animals were placed individually into a cylinder (40 cm height and 23 cm diameter), containing water up to 25 cm below the top. The immobility time was monitored for 5 min, after an initial 1 min adaptation period. The reduction of this immobility time is suggestive of an antidepressive-like action. This is a rodent behavioral test used for evaluation of antidepressant efficacy of new compounds and of experiments aimed at rendering or preventing depressive-like states [21, 22].

### Neurochemical assays

#### Measurements of striatal dopamine (DA) and DOPAC contents by HPLC

Degeneration of the dopaminergic nigrostriatal system causes dopaminergic depletion responsible for most of the motor symptoms of Parkinson’s disease [23]. Striatal DA and DOPAC levels were measured in 10% homogenates prepared in 0.1 M HClO_4_. After sonication for 30 s, the homogenates were centrifuged under refrigeration (15 min at 20,000 x g), the supernatant was filtered (0.2 μm, Millipore), and samples (20 μL, each) were injected into a high-performance liquid chromatography (HPLC) column (C18, 5 μm, 250 × 4.6 mm). The mobile phase was 0.163 M citric acid, pH 3.0, containing 0.02 mM EDTA, with 0.69 mM sodium octanesulfonic acid (SOS), as ion pairing reagent, 4% *v*/*v* acetonitrile and 1.7% *v*/*v* tetrahydrofuran. The concentrations of DA and DOPAC were determined by comparison with standards, and the results were expressed as ng/g tissue.

#### Determination of striatal nitrite contents

In this assay, the Griess reagent (1 part 0.1% naphthylethylenediamine dihydrochloride in distilled water plus 1 part 1% sulfanilamide in 5% H_3_PO_4_) indicates the presence of nitrites in the sample. Striatal homogenates (10% in KCl buffer) were centrifuged (10,000 x g for 10 min), and 100 μL supernatants were added to 100 μL Griess reagent. This mixture stayed on RT for 10 min. The standard NaNO_2_ curve was obtained (in spectrophotometer, at 520 nm) and used for calculating the results expressed as micromole nitrite per gram tissue [24].

#### Determination of striatal lipid peroxidation (tiobarbituric acid reactive substances, TBARS assay)

Lipid peroxidation expresses oxidative stress induced by ROS reactivity and a largely used method for that is the determination of malondihaldehyde (MDA) in biological samples [25]. The lipid peroxidation products are MDA and 4-hydroxy-2-nonenal (4-HNE), of which MDA is considered a good biomarker of oxidative stress and an end product of lipid peroxidation [26]. Striatal homogenates (10%) in 1.15% KCl were added (250 μL) to 1 mL 10% TCA followed by addition of 1 mL 0.6% thiobarbituric acid. After agitation, this mixture was maintained in a water-bath (95-100 °C) for 15 min. Then, the mixture was cooled on ice and centrifuged (1,500 x g/5 min). The TBARS content was determined in a plate reader, at 540 nm, with results expressed in micromole MDA per gram tissue. A standard curve with several MDA concentrations was also performed.

#### Immunohistochemistry assays for tyrosine hydroxylase (TH), dopamine transporter (DAT), VD3 receptor (VD3R), and tumor necrosis factor alpha (TNF-alpha) in rat striata

Dopaminergic neurons can be characterized by expression of tyrosine hydroxylase (TH), the rate-limiting enzyme in the catecholamine synthesis pathway [27, 28]. The physiological role of DAT is the re-uptake of released DA into presynaptic DA-terminals. DAT density has been reported to be downregulated in response to dopaminergic lesions, presumably to maintain synaptic dopamine levels [29]. The actions of VD3 are mediated by the vitamin D receptor (VD3R), a ligand-activated transcription factor that functions to control gene expression [11]. Furthermore, evidences indicate the participation of neuroinflammation mediators as TNF-alpha in the pathogenesis of PD [30]. For the immunohistochemical assays, coronal slices of striata were fixed in formaldehyde for 24 h followed by 70% alcohol immersion. Striatal sections were cut (5 μm), placed on slides, and washed three times with PBS followed by the addition of 3% hydrogen peroxide in PBS. Then, the primary antibodies anti-TH, anti-DAT, anti-VD3R, or anti-TNF-alpha were added after dilution, according to the manufacturers' instructions, in 0.05 M Tris buffer, pH 7.2–7.6, containing 1% BSA, and stayed overnight. The next day, the slices were washed two times with PBS followed by the addition of the secondary antibody (yellow reagent or Link, DAKO Cytomation) for 1 h in a cold chamber. Then, after washing again with PBS, the slices were soaked in streptavidin-peroxidase (red reagent, DAKO Cytomation) for 40 min. After another wash, a DAB solution (3-3-diaminobenzidine tetrahydrochloride, prepared according to the manufacturer’s instructions) was applied on the top of the slices, for 30 s, and these were mounted on a free xylol medium, for light microscopy analyses.

### Statistical analyses

All data were expressed as mean ± SEM. For all parameters, a one-way ANOVA was used followed by the Tukey’s post hoc test. The threshold for statistical significance was set to *p* < 0.05. All statistical analyses were performed with the GraphPad Prism (Version 6.0). For the immunohistochemical data, the optical density was determined by the Image J software (NIH, USA).

## Results

### Behavioral tests

**Fig. 1:**
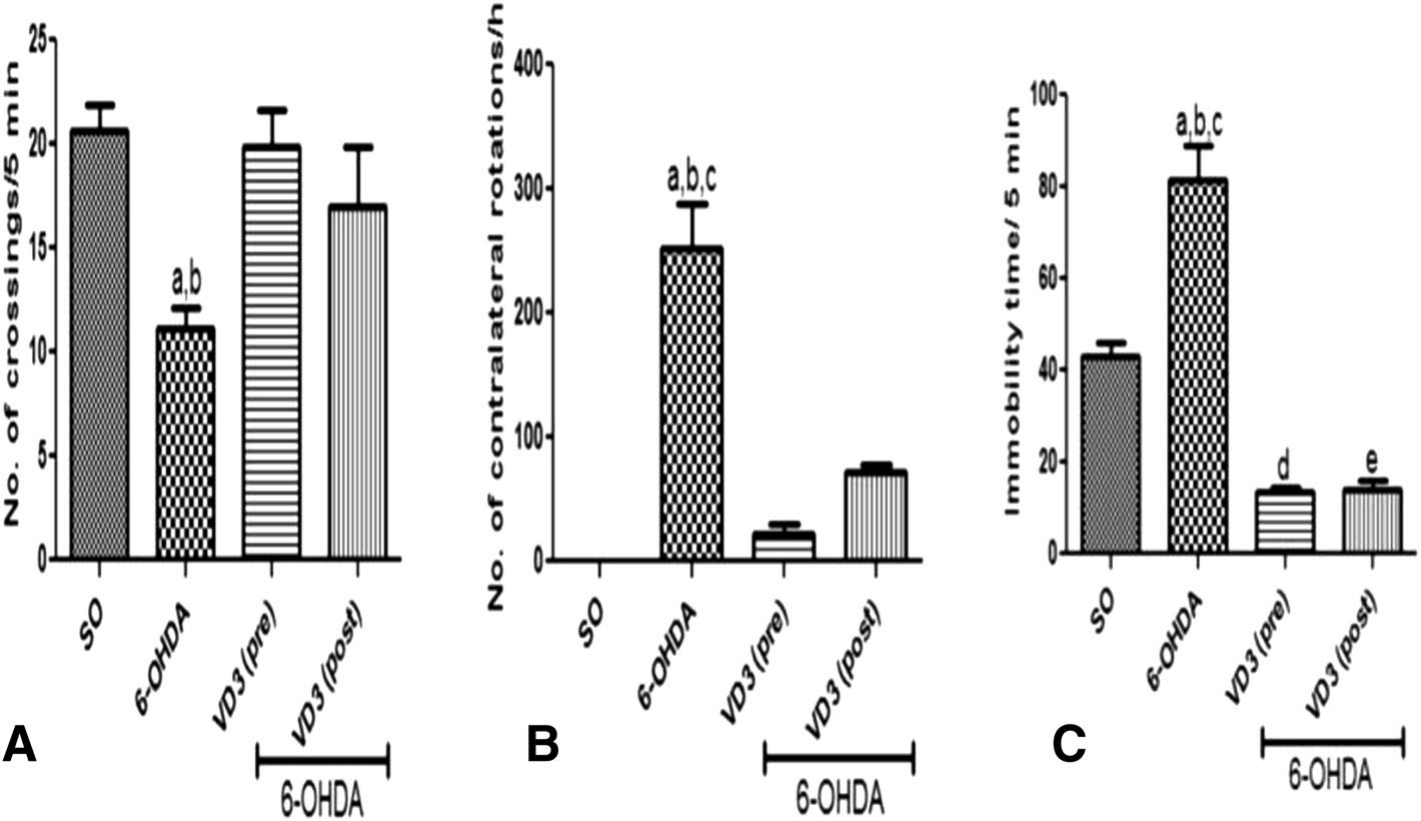
The pre- and post-treatments with vitamin D (VD3, 1 μg/kg, p.o.) reversed the increased number of crossings (**A**), the apomorphine-induced circling behavior (**B**), and the immobility time (**C**), compared with the non-treated 6-OHDA group. **A**: **a** vs. SO, *q* = 4.608, *p* < 0.05; **b** vs. 6-OHDA+VD3 (pre), *q* = 4.375, *p* < 0.05. **B**: **a** vs. SO, *q* = 13.53, *p* < 0.001; **b** vs. 6-OHDA+VD3 (pre), *q* = 12.37, *p* < 0.001; **c** vs. OHDA+VD3 (post), *q* = 9.673, *p* < 0.001. **C:** **a** vs. SO, *q* = 8.873, *p* < 0.001; **b** vs. 6-OHDA+VD3 (pre), *q* = 15.76, *p* < 0.001; **c** vs. 6-OHDA+VD3 (post), *q* = 15.63, *p* < 0.001; **d** vs. SO, *q* = 6.887, *p* < 0.001; **e** vs. SO, *q* = 6.754, *p* < 0.001 (one-way ANOVA and Tukey as the post hoc test)

In the open field test, the non-treated 6-OHDA group showed a reduced motor activity with a 46% decrease in the number of crossings/5 min. On the contrary, no significant differences were observed in the 6-OHDA group after VD3 treatments, relatively to the SO group. As a whole, treatments with VD3 showed a significant improvement on the animal’s mobility (Fig. 1a). In the apomorphine-induced circling behavior, while the non-treated 6-OHDA group showed 252 contralateral rotations/h, compared with that of the SO group, only 21 and 72 contralateral rotations/h were presented by the 6-OHDA groups after VD3 pre- and post-treatments, respectively (Fig. 1b). Importantly, the significant motor asymmetry observed in the non-treated 6-OHDA group, after apomorphine administration, validates the striatal lesion experimental procedure. In the forced swimming test, the non-treated 6-OHDA group showed an almost two times increase in immobility time, compared with the SO group, indicating a depressive-like behavior. Interestingly, the VD3 treated 6-OHDA groups showed values significantly lower than those of the SO group (Fig. 1c).

### Neurochemical assays

#### Dopamine (DA) and DOPAC determinations in the rat lesioned striata

**Fig. 2:**
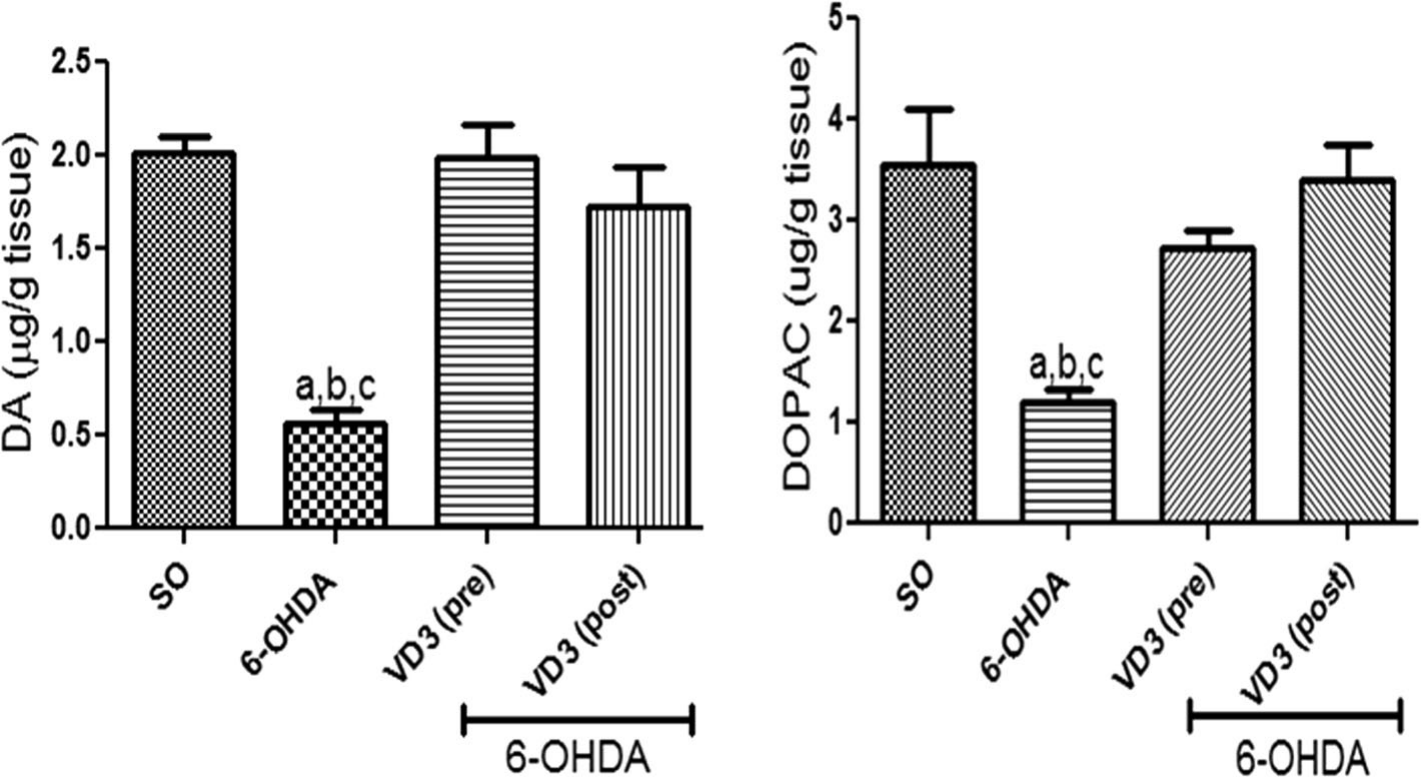
The pre- and post-treatments with vitamin D (VD3, 1 μg/kg, p.o.) reversed DA and DOPAC decreases, demonstrated in the striatal lesioned side of the non-treated 6-OHDA group. **DA**: **a** vs. SO, *q* = 10.16, *p* < 0.001; **b** vs. 6-OHDA+VD3 (pre), *q* = 10.45, *p* < 0.001; **c** vs. 6-OHDA+VD3 (post), *q* = 8.166, *p* < 0.001. **DOPAC:** **a** vs. SO, *q* = 7.290, *p* < 0.001; **b** vs. 6-OHDA+VD3 (pre), *q* = 5.40, *p* < 0.01; **c** vs. 6-OHDA+VD3 (post), *q* = 8.072, *p* < 0.001 (one-way ANOVA and Tukey as the post hoc test)

A 72% reduction in DA striatal contents was observed in the non-treated 6-OHDA group, compared with the SO group. Importantly, no significant alterations were demonstrated in DA levels, in the striata of 6-OHDA groups after VD3 treatments. Additionally, decreases (54%) were observed in striatal DOPAC contents, in the non-treated 6-OHDA group. Also, DOPAC values in both VD3-treated groups went towards those of the SO groups (Fig. 2).

#### Nitrite and lipid peroxidation measurements in the rat lesioned striata

**Fig. 3:**
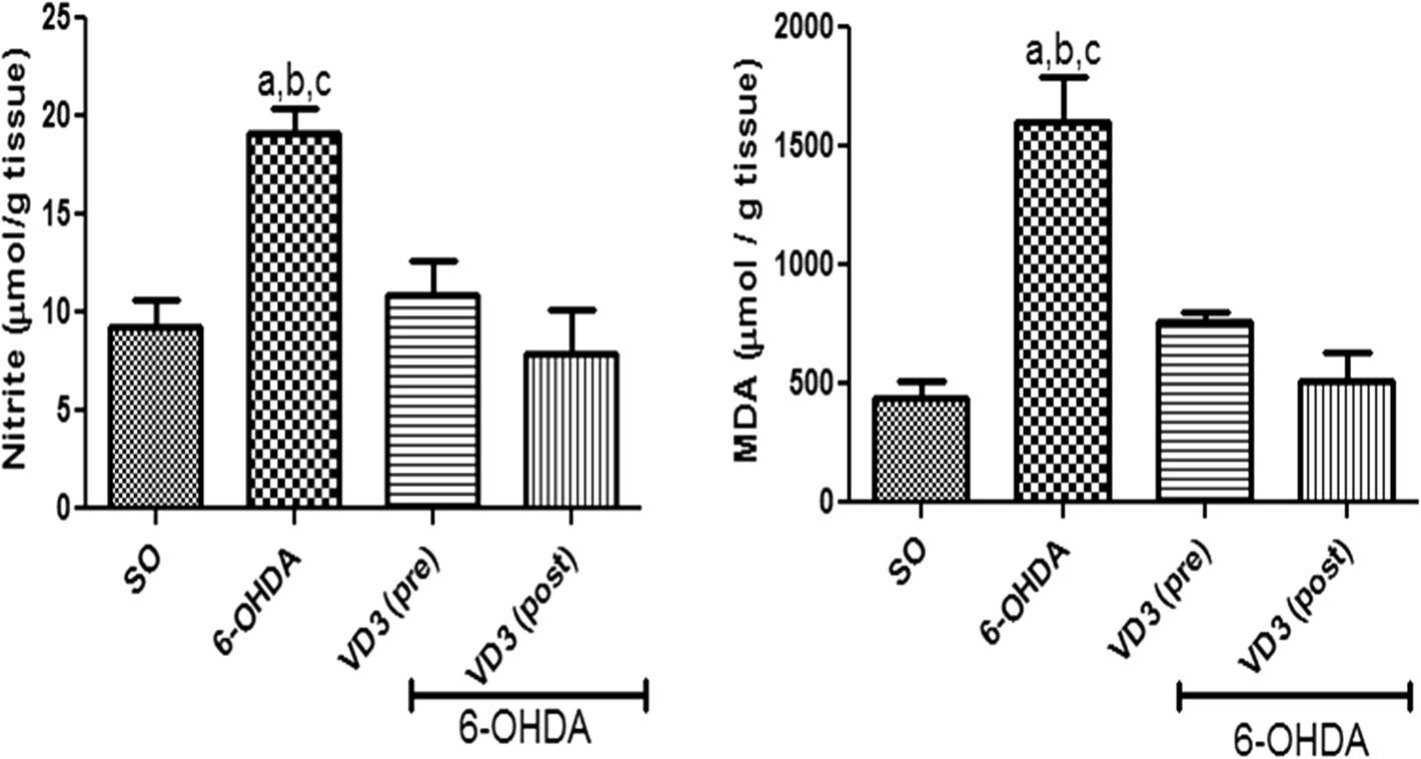
The pre- and post-treatments with vitamin D (VD3, 1 μg/kg, p.o.) reversed the increased nitrite contents and lipid peroxidation (TBARS), observed in the striatal lesioned side of the non-treated 6-OHDA animals. **Nitrite:** **a** vs. SO, *q* = 6.196, *p* < 0.01; **b** vs. 6-OHDA+VD3 (pre), *q* = 4.963, *p* < 0.05; **c** vs. 6-OHDA+VD3 (post), *q* = 6.120, *p* < 0.01. **TBARS:** **a** vs. SO, *q* = 11.04, *p* < 0.001; **b** vs. 6-OHDA+VD3 (pre), *q* = 7.416; *p* < 0.001; **c** vs. 6-OHDA+VD3 (post), *q* = 9.040, *p* < 0.001 (one-way ANOVA and Tukey as the post hoc test)

A two times increase in striatal nitrite contents was observed in the non-treated 6-OHDA group, compared with the SO group. On the contrary, no significant changes were seen in the 6-OHDA group, after VD3 treatments, whose values were not different from those of the SO groups. Increases in the striatal lipid peroxidation of almost four times were observed in the non-treated 6-OHDA group, related to the SO group. However, no changes were seen in the 6-OHDA group after pre- or post-VD3 treatments (Fig. 3).

#### Immunohistochemistry for TH, DAT, TNF-alpha, and VD3R in the rat lesioned striata

**Fig. 4:**
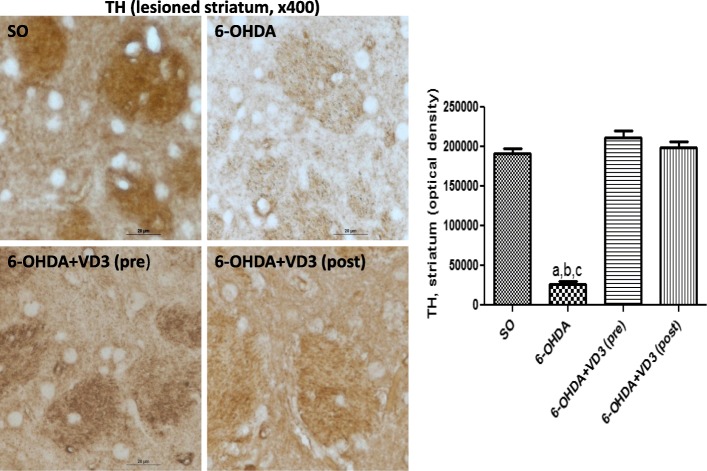
The pre- and post-treatments with vitamin D (VD3, 1 μg/kg, p.o.) reversed the decreased immunostaining for tyrosine hydroxylase (TH), observed in the striatal lesioned side of the 6-OHDA group. **a** vs. SO, *q* = 20.80, *p* < 0.001; **b** vs. 6-OHDA+VD3 (pre), *q* = 24.66, *p* < 0.001; **c** vs. 6-OHDA+VD3 (post), *q* = 22.92, *p* < 0.001 (one-way ANOVA and Tukey as the post hoc test)

**Fig. 5:**
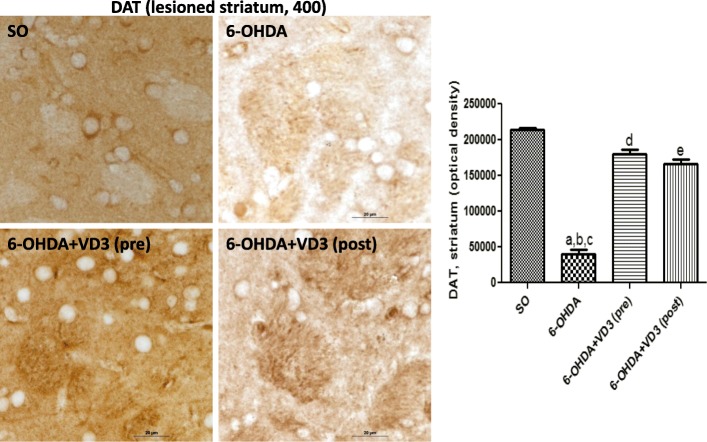
The pre- and post-treatments with vitamin D (VD3, 1 μg/kg, p.o.) partly reversed the decreased immunostaining for the dopamine transporter (DAT), observed in the lesioned striata of the non-treated 6-OHDA rats. **a** vs. SO, *q* = 31.29, *p* < 0.001; **b** vs. 6-OHDA+VD3 (pre), *q* = 25.24, *p* < 0.001; **c** vs. 6-OHDA+VD3 (post), *q* = 19.81, *p* < 0.001; **d** vs. SO, *q* = 6.046, *p* < 0.001; **e** vs. SO, *q* = 7.285, *p* < 0.001 (one-way ANOVA and Tukey as the post hoc test)

**Fig. 6:**
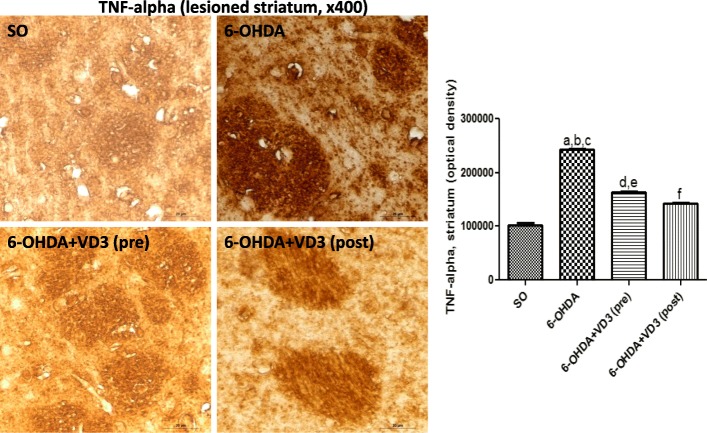
The pre- and post-treatments with vitamin D (VD3, 1 μg/kg, p.o.) partly reversed the TNF-alpha immunostaining, observed in the lesioned striata of non-treated 6-OHDA animals. **a** vs. SO, *q* = 55.10, *p* < 0.001; **b** vs. 6-OHDA+ VD3 (pre), *q* = 31.35, *p* < 0.001; **c** vs. 6-OHDA+VD3 (post), *q* = 39.21, *p* < 0.001; **d** vs. SO, *q* = 23.74, *p* < 0.001; **e** vs. 6-OHDA+VD3 (post), *q* = 15.89, *p* < 0.001; **f** vs. 6-OHDA+VD3 (pre), *q* = 7.857, *p* < 0.001 (one-way ANOVA and Tukey as the post hoc test)

**Fig. 7:**
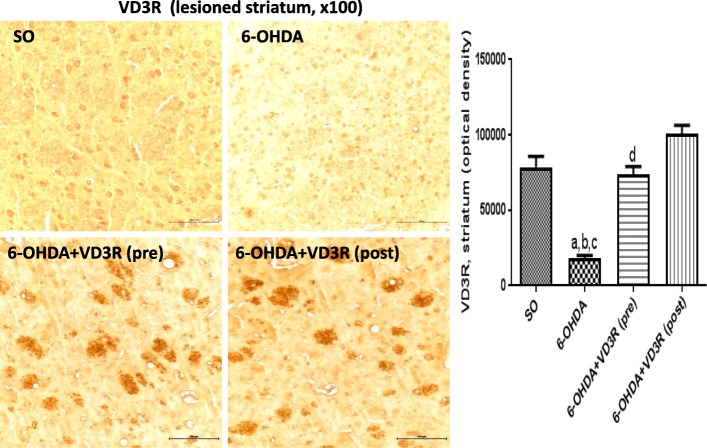
The pre- and post-treatments with vitamin D (VD3, 1 μg/kg, p.o.) partly reversed the VD3 receptor (VD3R) immunostaining, observed in the lesioned striata of the non-treated 6-OHDA animals. **a** vs. SO, *q* = 11.38, *p* < 0.001; **b** vs. 6-OHDA+VD3 (pre), *q* = 10.48, *p* < 0.001; **c** vs. 6-OHDA+VD3 (post), *q* = 15.49, *p* < 0.001; **d** vs. 6-OHDA+VD3 (post), *q* = 5.009, *p* < 0.05 (one-way ANOVA and Tukey as the post hoc test)

The immunohistochemistry data for TH revealed an 86% decrease in the non-treated 6-OHDA groups, compared with the SO groups. Interestingly, no reduction was observed in the 6-OHDA groups, after pre- and post-VD3 treatments (Fig. 4). Similarly, the non-treated 6-OHDA showed an 81% decrease in DAT immunostaining, relatively to the SO groups. The decreases in DAT values were much lower in the 6-OHDA groups, after pre- and post-VD3 treatments (16 and 22% decreases, respectively), relatively to the SO groups (Fig. 5). The TNF-alpha data presented a 2.4 times increase in immunostaining for this pro-inflammatory cytokine, in the non-treated 6-OHDA group, in relation to the SO group. This value dropped to 1.6 and 1.4 times increases after pre- and post-treatments with VD3, respectively (Fig. 6). A significant decrease (77%) in the striatal VD3R immunoreactivity was demonstrated in the non-treated 6-OHDA group, compared with the SO group. No significant changes were noticed in the striata from both VD3 pre- or post-treatment groups (Fig. 7).

## Discussion

VD3 has been shown to have a close relationship to brain function and evidences for that have significantly increased in the last decades. Although some clinical studies support a role for VD3 neuroprotection in PD [31–35], other studies [36] show no clear causal association between lower VD3 concentration and increased risk for PD. In the present study, we showed that VD3 treatments reversed partially or completely the behavioral, neurochemical, and immunohistochemical alterations, as those observed in the 6-OHDA-induced PD model, in rats. Thus, we observed that VD3 treatments resulted in the attenuation of the apomorphine circling behavior, which correlates positively to the degree of 6-OHDA striatal lesion [20].

Some studies have shown the relationship between VD3 deficiency and motor impairment, associated with a decrease in the number of crossings and rearing in the open field test [37], what corroborates with our results, where VD3 supplementation caused an improvement in the animals’ motor coordination. Furthermore, VD3 completely reversed the increased immobility time demonstrated in the non-treated 6-OHDA group, indicating an antidepressive-like behavior. VD3 deficiency has been related with a reduced immobility time, on the forced swimming test [38]. Low VD3 concentrations in the blood seem to be associated with adult depression and postpartum depression [39, 40]. In addition, the correction of VD3 deficiency or its supplementation may be effective for depression states, as observed in randomized clinical trials [41, 42].

VD3 is known to regulate some factors involved with the ontogeny of the dopaminergic system. Thus, neonatal rats whose mothers were deprived of VD3, presented a decreased DA turnover [43]. In addition, VD3 deficiency resulted in altered dopaminergic metabolism in the rat forebrain, with a decreased conversion of DOPAC to HVA [44]. Evidences indicate that VD3 can not only reduce the extent of dopaminergic denervation, but also offer functional benefit to animals subjected to a Parkinson’s disease model [45]. An alteration in the development of the dopaminergic system and on dopamine-mediated behaviors has also been shown in female VD3-deficient rats [44].

A range of studies showed that the *substantia nigra* presents one of the highest brain concentrations of VD3 receptor (VD3R), indicating the importance of this mediator in dopaminergic pathways [46]. This suggestion was supported by the results obtained on the rotational test, since the animals receiving VD3 did not present motor stereotypy that characterizes the unilateral dopamine depletion. Tyrosine hydroxylase (TH), a rate-limiting enzyme for dopamine synthesis, might be directly modulated by VD3, as observed by immunohistochemistry staining. Previous studies [47] have shown that VD3 administration, before or after the local 6-OHDA injury, partially restores TH protein and TH-immunoreactive fibers in the striatum and *substantia nigra.* These effects also increased the glial derived neurotrophic factor (GDNF) protein, contributing to VD3 neuroprotective effects on dopaminergic neurons. Data from rat mesencephalic cultures of dopaminergic neurons point out to a dose-dependent increase in the number of dopaminergic neurons and upregulation of GDNF, after the addition of VD3 [45].

Several studies have examined the protective effects of VD3 against dopaminergic toxins, as 6-OHDA, showing that VD3 restored partially both nigral and striatal DA levels and also the TH immunostaining in positive cells [48–50], results that are comparable to ours. The mechanism by which VD3 promotes recovery of dopaminergic release and content remains to be determined, but evidences indicated its potential for affecting calcium metabolism, apoptosis, inflammation, immunomodulation, detoxification, and upregulation of neurotrophins [51–53].

Evidences also suggest that oxidative stress plays a major role in neurodegenerative diseases, including PD [39, 54, 55]. Not only DA metabolism could contribute to that, but also mitochondrial dysfunction, leading to an increase in ROS and activated microglia, and producing NO and ROS during neuroinflammatory responses. The VD3 treatment was shown to increase cell viability and to decrease ROS production, in cone cells under oxidative stress [56]. Furthermore, VD3 deficiency induced ROS production by neutrophil, in spontaneous hypertensive rats [57], and prevented oxidative stress in adipose tissue of diabetic mice [58]. These data agree with ours, showing that VD3 treatments significantly decreased nitrite contents and lipoperoxidation in lesioned striata, as related to the same area of the non-treated 6-OHDA rats, indicating that VD3 presents an antioxidant action.

It is known that, besides the reduction of immunostaining for TH, the reduction of dopaminergic neurons in PD patients and animal models of parkinsonism leads to substantial loss of the presynaptic markers, such as the dopamine transporter (DAT), probably due to compensatory effects that aim to maintain synaptic functionality in the face of decreased dopamine [59–61]. Nevertheless, in experiments with toxins that induce neuronal death, DAT-deficient immunostaining is a direct reflection of the installed neurodegenerative process [62, 63]. Thus, DAT staining can be considered an indicator of membrane integrity of dopaminergic neurons.

As expected, in the present work, the striata of the 6-OHDA rats showed a decrease in DAT immunostaining, which was also reversed although to a lower extent. A DAT blockade is observed with some antidepressant drugs [50], and VD3 significantly decreased the immobility time, suggesting an antidepressive-like behavior. Depressive behavior is a common feature of Parkinson’s disease patients [64], and clinical studies are consistent with the hypothesis that low VD3 concentration is associated with depression [65]. How VD3 acts to reduce depression is unclear. Evidences indicate that VD3 acts by reducing the increased neuronal levels of Ca^2+^ associated with depression [66].

All the results presented so far converge to the neuroprotective potential of VD3. Most of its biological actions are now exerted through the nuclear VD3 receptor (VD3R)-mediated control of target genes. VD3R is present in mammalian brain and by proteomic techniques was also shown to be present in the adult rodent brain. However, its expression is quantitatively lower in the brain, comparatively with the gut and kidneys from both embryonic and adult tissues [67]. VD3R belongs to the nuclear hormone receptor superfamily, which also includes receptors for cortisol and thyroid hormone, and acts as a ligand-inducible transcription factor [68, 69]. In the present study, we showed that VD3 treatments increase the immunoreactivity for VD3R, in the striata from non-treated 6-OHDA animals. Furthermore, VD3 treatments activated VD3R expression and attenuated neurological deficits observed in a traumatic brain injury model [70].

VD3 neuroprotective effects involve several independent mechanisms [71] and, among them, the potent immunomodulatory activities in both innate and adaptive immunity [72]. In the last decades, neuroinflammation has been considered an important cause for progression of PD [73, 74]. In the MPTP-induced PD model, researchers demonstrated that the treatment with VD3 attenuates iNOS and pro-inflammatory cytokines expression [75]. The same pattern could be observed in animal models using the 6-OHDA neurotoxin. A post-mortem study showed the increased levels of TNF-α and other inflammatory mediators in the brain of Parkinson’s disease patients [76]. VD3 was shown to significantly attenuate the loss of TH-positive neuronal cells, microglial cell activation, iNOS expression, among other typical hallmarks of microglia activation, in an animal model of PD similar to ours [75].

Protein misfolding plays a key role in neurodegenerative diseases and is characterized in PD by the accumulation of intraneuronal aggregates of α-synuclein, main component of Lewy bodies. In addition, evidence indicates that α-synuclein is a prion-like protein and therefore PD is considered to be a prion-like disease [77–80]. Furthermore, the transcellular propagation of protein aggregation might underlie the progression of neurodegenerative diseases, and α-synuclein fibrils are able in PD to spread via cell-to-cell transfer [80, 81]. Most importantly, the cellular form of the prion protein (PrPc) acts as a receptor for α-synuclein fibrils [80]. Curiously, Vitamin D2 (ergocalciferol) has been shown to interact with the human PrPc and may be a suitable agent to target PrPc in the brain and, therefore, a potential therapeutic candidate for prion diseases [82].

## Conclusion

Recently (see Additional file 1), we observed significant effects of VD3 (0.5 and 1.0 μg/kg, p.o.) after a 7-day treatment in the formalin test of nociception and in the carrageenan-induced paw edema test in mice. We demonstrated that the VD3 treatments inhibited only the second phase of the formalin test, associated with an inflammatory response. In addition, VD3 reduced the edema volume at the 3rd hour that corresponds to a maximal effect. These data are related to the VD3 inhibition of prostaglandin synthesis (second phase of the formalin test) and to the reduced expression of inflammatory mediators such as COX-2 and iNOS (carrageenan-induced paw edema test). In addition, these results give support to the participation of the anti-inflammatory action of VD3 on its neuroprotective effect.

Therefore, all together, the neuroprotective action of VD3 as demonstrated in the present study, is probably due to its anti-inflammatory and antioxidant properties. VD3 signaling is involved with neurocognitive decline and neurodegenerative diseases. Most importantly, almost all VD3 actions are mediated by the VD3R [83], pointing out to the role of VD3R in the neuroprotective effects of VD3. VD3 has been shown to downregulate the L-type voltage-sensitive calcium channels and to upregulate nerve growth factor. The suppression of VD3R interrupts these events and neurons become more vulnerable to neurodegeneration [84]. Furthermore, VD3, by its anti-inflammatory and antioxidant properties, by its actions on calcium channels and growth factors and, mainly, by increasing the brain VD3R expression, is a strong candidate for submission to well-designed clinical trials, proposing this steroid hormone for prevention or treatment of neurodegenerative diseases as PD.

## Additional file

Additional file 1: Evaluation of the effects of Vitamin D (VD3) on the formalin and carrageenan-induced paw edema tests, after 7-day treatments. (DOCX 131 kb)

## Abbreviations

6-OHDA: 6-hydroxydopamine
AD: Alzheimer’s disease
BSA: Bovine serum albumin
DA: Dopamine
DAB: (3,3′-diaminobenzidine)
DAT: Dopamine transporter
DOPAC: 3,4-Dihydroxyphenylacetic acid
GDNF: Glial cell-derived neurotrophic factor
iNOS: Inducible nitric oxide synthase
MDA: Malondialdehyde
PBS: Phosphate buffered saline
PD: Parkinson’s disease
ROS: Reactive oxygen species
*SNpc*: *Substantia nigra pars compacta*
TBARS: Tiobarbituric acid reactive substances
TH: Tyrosine hydroxylase
TNF-alpha: Tumor necrosis factor alpha
VD3: 1,25-dihydroxyvitamin D3
VD3R: Vitamin D3 receptor
VD3R/RXR: Vitamin D3 nuclear receptor complex with the retinoid X receptor
