# Supplementary material for: Vitamin D protects dopaminergic neurons against neuroinflammation and oxidative stress in hemiparkinsonian rats
Source: J Neuroinflammation. 2018 Aug 31;15:249. doi: 10.1186/s12974-018-1266-6 (PMC6119240; doi:10.1186/s12974-018-1266-6)
Supplement: Supplementary file 1 — Evaluation of the effects of Vitamin D (VD3) on the formalin and carrageenan-induced paw edema tests, after 7-day treatments. (DOCX 131 kb) [file 12974_2018_1266_MOESM1_ESM.docx]

**Additional file 1:** Evaluation of the effects of Vitamin D (VD3) on the formalin test and on the carrageenan-induced paw edema test, in mice after 7-day treatments

*Formalin test in mice*

This is considered a valid and reliable model of nociception sensitive for analgesic drugs. There are two distinct periods of paw licking activity, each one measured for 5 min. The early phase (1^st^ phase) lasts the first 5 min and the late phase (2^nd^ phase), from 20-30 min after the subplantar injection of 1% formalin. The first phase is considered a direct effect on nociceptors and represents the neurogenic phase. The 2^nd^ phase represents the inflammatory response (Hunskaar and Hole, 1987, Tjolsen et al., 1992). Morphine, used as reference, significantly reduces the responses in the two phases of the formalin test. In our study, male Swiss mice (25 g) were previously treated by gavage with Vit. D (0.5 or 1.0 µg/kg, p.o., 7 days). The control group was treated with the vehicle (1% DMSO in an aqueous solution) for the same period of time. After that, the animals were subjected to the formalin test. We showed that while morphine reduced in 83% the licking time (s) in the 1^st^ phase, no change was noticed after the VD3 treatments, compared with the control group. However, decreases of 60 and 54% in the licking time were demonstrated in the 2^nd^ phase of the formalin test, after the VD3 treatments with the doses of 0.5 and 1.0 µg/kg, respectively. Morphine caused a 91% reduction in the 2^nd^ phase. These results strongly support the effect of Vit. D on the inflammatory response (2^nd^ phase), evaluated by the formalin test (Fig. 1).

| **** | **** |
| --- | --- |

Fig. S1. VD3 (0.5 and 1.0 µg/kg, p.o., 7 days) significantly reduced only the responses of the 2^nd^ phase (inflammatory) of the formalin test in mice. Morphine was used as reference. 1^st^ phase: a. vs. Control, p<0.001; b. vs. VD3 0.5, p<0.001; c. vs. VD3, p<0.001. 2^nd^ phase: a. vs. Control, p<0.001; b. vs. Control, p<0.001; c. vs. Control, p<0.01 (One way ANOVA and Tukey, multiple comparison test).

*Carrageenan-induced paw edema in mice*

The subplantar injection of 1% carrageenan to mice induces an acute swelling that becomes maximal 3-5 h after the injection and subsides by 24 h (Winter et al., 1962; Posadas et al., 2004). This acute inflammation model can be used to assess the production of inflammatory mediators, at the site of inflammation, and the properties of anti-inflammatory drugs (Fehrenbacher et al., 2012). In the present study, male Swiss mice (25 g) were orally treated by gavage with VD3 (0.5 and 1.0 µg/kg, 7 days). After treatments, the animals received a subplantar injection of carrageenan and the edema volume (mL) was measured before the carrageenan injection (T0) and after 3 h (T3) by a plethysmography apparatus (Ugo Basili, Italy). The control group was also orally treated with the vehicle (1% DMSO in an aqueous solution), for the same period of time. The results are expressed as the difference between T3 and T0. Our results showed 50 and 56% reductions in the edema volume at the 3^rd^ h (T3) after the carrageenan injection, compared with the control group at the same period of time.

Fig. S2. VD3 (0.5 and 1.0 µg/kg, p.o., 7 days) significantly reduced the edema volume (mL), at the 3^rd^ h after the subplantar injection of carrageenan in mice. a. vs. Control, p<0.001; b. vs. Control, p<0.001 (One way ANOVA and Tukey, multiple comparison test).

In conclusion, our results show a significant anti-inflammatory effect of VD3, after 7- day treatments, that probably involves the inhibition of prostaglandin synthesis (2^nd^ phase of the formalin test) and inflammatory mediators, as COX-2 and iNOS (carrageenan-induced paw edema test).

**References**

Fehrenbacher, J.C., Vasko, M.R., Duarte, D.B. 2012. Models of inflammation: carrageenan- or complete Freund’s adjuvant-induced edema and hypersensitivity in the rat. Curr Protoc Pharmacol 2012 March; 0.5: Unit5.4. doi:10.1002/0471141755.ph0504s56.

Hunskaar, S., Hole, K. 1987. The formalin test in mice: dissociation between inflammatory and non-inflammatory pain. Pain 30: 103-114.

Posadas, I., Bucci, M., Roviezzo, F. et al. 2004. Carrageenan-induced mouse paw oedema is biphasic, age-weight dependent and displays differential nitric oxide cyclooxygenase expression. Br J Pharmacol 142: 331-338.

Tjolsen, A., Berge, O.G., Hunskaar, J.H. et al. 1992. The formalin test: an evaluation of the method. Pain51: 5-17.
